# Functional Mobility as a Prognostic Marker in Geriatric Oncology: A Prospective Study Using Gait Speed

**DOI:** 10.3390/medicina62091815

**Published:** 2026-09-21

**Authors:** Beyza Ünlü, Hacer Demir, Sena Ece Davarcı, Yaşar Culha, Meltem Baykara

**Affiliations:** 1 Department of Medical Oncology, Afyonkarahisar Health Sciences University, Afyonkarahisar 03030, Turkey; 2 Department of Medical Oncology, Sivas Numune Hospital, Sivas 58050, Turkey

**Keywords:** geriatric oncology, gait speed, functional mobility, chemotherapy toxicity, overall survival, physical performance

## Abstract

*Background and Objectives*: Functional impairment is common in older adults with cancer and may influence treatment outcomes. This study aimed to evaluate the prognostic value of objectively measured functional mobility, with gait speed as the primary analytical variable, for survival, treatment response, and chemotherapy-related toxicity in geriatric cancer patients. *Materials and Methods:* In this single-center prospective study, 102 patients aged 65 years and older who were chemotherapy-naive were enrolled. Before treatment initiation, functional mobility was assessed using a 3-m gait-speed test at the patient’s usual pace. Gait speed was analyzed primarily as a continuous variable; the sample-median cutoff was retained for secondary descriptive analyses, and 0.8 m/s was evaluated in a sensitivity analysis. Associations with survival, treatment response, and toxicity were evaluated using survival and regression analyses. *Results:* The mean gait speed was 0.481 ± 0.176 m/s. Among patients with metastatic disease, higher continuous gait speed was associated with lower mortality in univariable analysis (HR = 0.752, 95% CI: 0.605–0.934, *p* = 0.010) and after adjustment for age, sex, and ECOG performance status (adjusted HR = 0.647, 95% CI: 0.490–0.853, *p* = 0.002). Gait speed was not significantly associated with metastatic PFS, non-metastatic OS or DFS, or grade ≥ 3 toxicity. The 0.8 m/s sensitivity analysis was limited by marked group imbalance. *Conclusions:* Higher baseline gait speed was associated with lower mortality in metastatic geriatric cancer patients, whereas statistically significant associations were not detected for PFS, non-metastatic survival outcomes, or grade ≥ 3 toxicity. These exploratory findings require validation in larger and more homogeneous cohorts.

## 1. Introduction

The global population is rapidly aging, and cancer incidence increases with age [1]. It is projected that by 2030, approximately 70% of all cancers will occur in adults aged 65 and older. This makes treatment decisions more difficult for oncologists in elderly patients with increased vulnerability to treatment-related toxicity [1]. Unlike younger patients, elderly cancer patients often have multiple comorbidities, polypharmacy, cognitive impairment, and decreased physiological reserves, increasing the risk of adverse outcomes during cancer treatment [2,3,4]. Currently widely used performance status scales, such as the Eastern Cooperative Oncology Group (ECOG) performance status, have been primarily developed in younger populations and may not adequately reflect the functional capacity status in geriatric patients [5]. This limitation has led to increasing interest in comprehensive geriatric assessment (CGA) to identify vulnerable elderly individuals and guide treatment decisions in geriatric oncology [6,7]. Comprehensive geriatric assessment: It is a holistic assessment method that includes numerous parameters such as functional status, comorbidity burden, cognitive function, nutritional status, psychological status, and social support [6,7]. Studies have shown that quantitative and repeatable assessments of functional capacity using physical performance tests such as walking speed and grip strength have predicted physiological reserve and the ability to tolerate cancer treatment [8,9,10,11]. Objective physical function measurements provide standardized, clinician-independent assessments and can be integrated into routine oncology practice [8,9].

Walking speed and gait tests are important components of CGA and play a critical role in determining functional capacity, especially in elderly cancer patients [8,9]. Walking speed, generally measured as the time it takes for an individual to walk a short distance (usually 3, 4, or 6 m) at their usual pace, is a simple and quick functional assessment tool [10,11]. Walking speed reflects the function of multiple physiological systems, including musculoskeletal strength, cardiovascular capacity, neurological coordination, and cognitive functions [10,12]. Clinical studies have shown that lower walking speed in the geriatric population is associated with an increased risk of hospitalization and mortality [10,13,14]. These results suggest that walking speed may have a similar prognostic role in older adults with cancer and may contribute to identifying patients at high risk for treatment-related complications and poor survival outcomes. Despite several prospective studies, evidence regarding the use of walking speed as a predictor of treatment outcomes in geriatric oncology is lacking [15,16,17]. Studies show significant heterogeneity in terms of cancer type, measurement protocols, patient selection, and outcome definitions [18,19]. Some studies have failed to demonstrate significant associations between walking speed and clinical outcomes [20]. These mixed findings raise important questions about the use of walking speed as a prognostic tool in different cancer types and treatments. Furthermore, the relationship between walking speed and individual treatment outcomes other than survival, such as treatment-related toxicity, dose adjustments, and quality of life, has not been sufficiently characterized. While some studies have shown that geriatric assessment tools involving physical performance predict non-fatal toxicities such as grade 3–4 infections, acute renal failure, and prolonged hospital stay during intensive chemotherapy, other studies have reported no additional predictive value [16,17]. Understanding whether functional scales such as walking speed can reliably predict not only survival but also treatment tolerance is critical for clinical use. In this study, walking speed was assessed using the 3-m walk test to predict treatment response and clinical outcomes in geriatric cancer patients, and the relationships between walking speed and overall survival and treatment-related toxicities were examined in different cancer populations. Furthermore, the study aimed to evaluate the additional prognostic value of walking speed over conventional performance measures (ECOG).

## 2. Materials and Methods

The study was designed as a single-center, prospective study. Patients aged 65 years and older who had not previously received chemotherapy underwent functional mobility assessment before the first course of chemotherapy. Two distinct mobility measures were available. For the Timed Up and Go (TUG) test, the patient sat upright in a standard-height (44–47 cm) chair, stood up, walked 3 m to a marked point, turned, walked back to the chair, and sat down; total TUG duration was recorded in seconds. Separately, gait speed was assessed during a 3-m walk at the patient’s usual pace and recorded in meters per second (m/s). Thus, gait speed was not calculated by converting the total TUG duration. Assistive devices were permitted when normally used, but no physical assistance was provided. Although TUG was recorded as part of the baseline functional assessment, it was not included in the present prognostic analyses; all analyses reported in this study were based on the separately measured 3-m gait speed.

Gait speed was the primary exposure for the prognostic analyses and was modeled continuously, with hazard ratios reported per 0.1 m/s increase. The sample median gait speed (0.462 m/s) was retained only for secondary descriptive and Kaplan–Meier analyses; an additional sensitivity analysis used the clinically recognized 0.8 m/s threshold. Patients were also characterized by age, ECOG performance status, sex and metastatic status. Treatment-related adverse events were monitored during systemic treatment and graded according to the Common Terminology Criteria for Adverse Events (CTCAE), version 5.0. Treatment response was assessed radiologically at the end of the planned treatment period according to RECIST version 1.1. OS denoted time to death from any cause; PFS denoted progression or death in metastatic disease; and DFS denoted recurrence/progression or death in non-metastatic disease. Patients without the relevant event were censored at their last available follow-up. Survival curves were estimated using Kaplan–Meier analysis and compared with the log-rank test. Cox models were deliberately parsimonious and included continuous gait speed, age, sex (female as the reference), and ECOG performance status (ECOG 0 as the reference). Cancer type was not included in these models because the number of tumor categories relative to subgroup size would substantially increase model complexity and the risk of overfitting. The proportional-hazards assumption for the primary metastatic OS gait-speed effect was assessed using a gait-speed-by-log(time) interaction. Grade ≥ 3 toxicity was evaluated using a parsimonious logistic regression model including gait speed, age, and sex because only 17 such events occurred. Categorical comparisons were considered exploratory in the setting of multiple toxicity outcomes. Statistical analyses were conducted using SPSS v20 (IBM Corp., Armonk, NY, USA), and two-sided *p* < 0.05 was considered statistically significant. No formal a priori sample-size calculation was performed.

## 3. Results

### 3.1. Patient Characteristics and Distribution of Gait Speed

**Figure 1:**
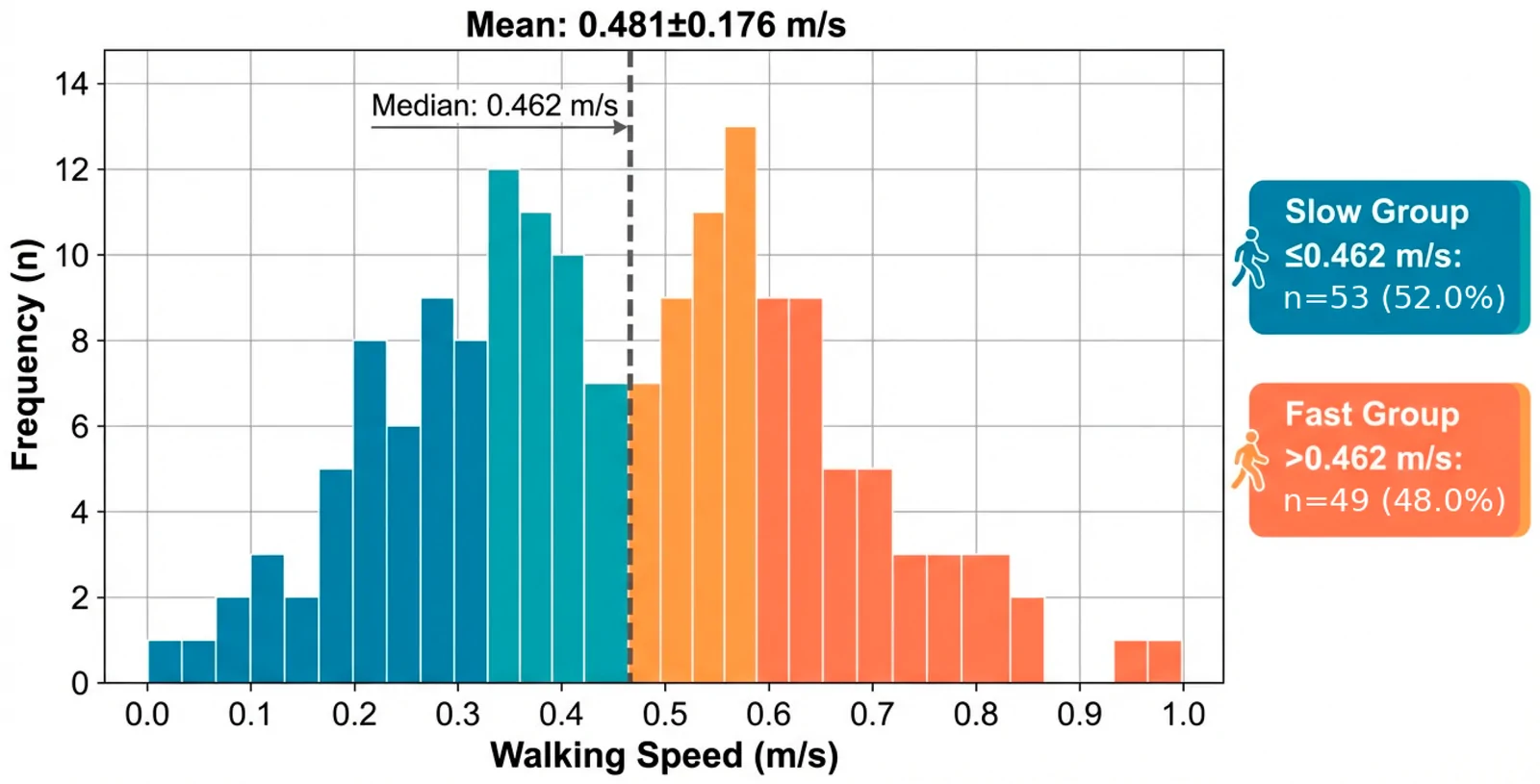
Distribution of Gait Speed in the Study Population. Distribution of gait speed among geriatric cancer patients based on the 3-m walking test. The median gait speed (0.462 m/s) was used as the cut-off value to categorize patients into slow and fast walking groups.

A total of 102 geriatric cancer patients were included in the study. The mean age of the patients was 71.0 ± 4.7 years (range: 65–86); 64.7% (n = 66) were male and 35.3% (n = 36) were female. The majority of the patients included in the study had an ECOG performance status of 1 (72.5%). The most common cancer types were lung and breast cancers. Baseline patient characteristics according to gait-speed group are presented in Table 1. Mean gait speed was 0.481 ± 0.176 m/s (range: 0.170–1.000 m/s). The median gait speed was calculated as 0.462 m/s; using this value for secondary descriptive grouping, 53 patients (52.0%) were classified as slow and 49 (48.0%) as fast (Figure 1). The median observed follow-up duration for the overall cohort was 6.42 months (range: 0.10–21.50 months). Among 50 patients with metastatic disease, 42 deaths and 43 PFS events were observed, corresponding to 8 and 7 censored observations for OS and PFS, respectively. Among 52 patients with non-metastatic disease, 29 deaths and 32 DFS events were observed, corresponding to 23 and 20 censored observations, respectively.

### 3.2. Survival Analyses and Treatment Response in Metastatic Patients

**Figure 2:**
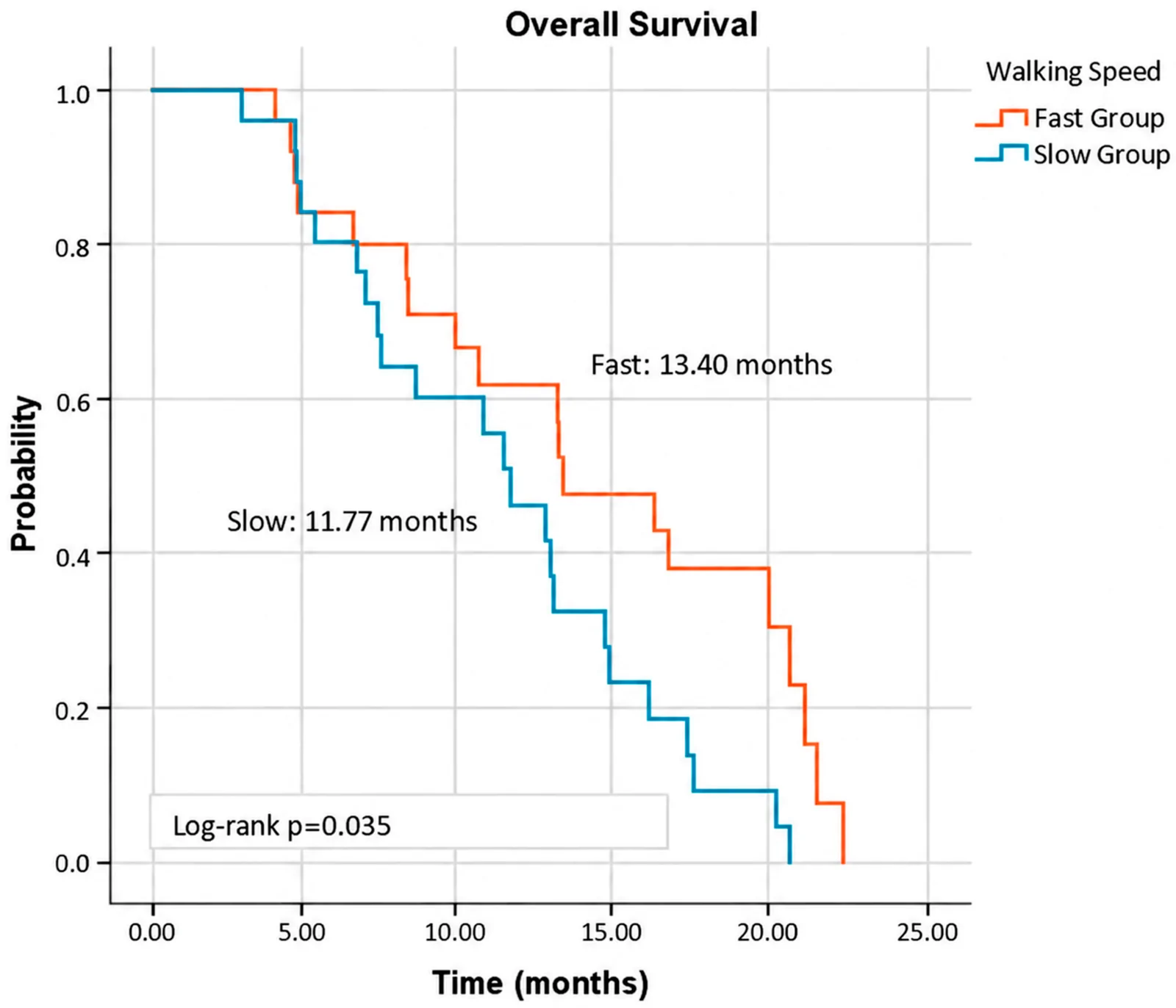
Kaplan–Meier Overall Survival Curves in Metastatic Patients According to Gait Speed. Kaplan–Meier curves demonstrating overall survival (OS) in metastatic patients stratified by gait speed. Patients in the fast-walking group showed significantly longer median survival compared to the slow-walking group (*p* = 0.035, log-rank test).

In the Kaplan–Meier survival analysis, median survival times were evaluated according to ECOG performance status, sex, smoking status, presence of chronic disease, recent cancer-related surgery history, and gait speed. Median overall survival was 13.4 months in the fast-walking group and 11.7 months in the slow-walking group, and this difference was statistically significant (*p* = 0.035) (Figure 2). Although longer survival durations were observed in female patients compared to males and in non-smokers compared to smokers, these differences were not statistically significant. Similarly, higher survival durations were observed in individuals without chronic disease and those without a history of surgery (Table 2).

In the primary Cox analyses, gait speed was modeled continuously. Among metastatic patients, each 0.1 m/s increase in gait speed was associated with a lower hazard of death in univariable analysis (HR = 0.752, 95% CI: 0.605–0.934, *p* = 0.010) and remained significant after adjustment for age, sex, and ECOG performance status (adjusted HR = 0.647, 95% CI: 0.490–0.853, *p* = 0.002). Continuous gait speed was not significantly associated with PFS in univariable analysis (HR = 0.846, 95% CI: 0.703–1.017, *p* = 0.075) or adjusted analysis (HR = 0.838, 95% CI: 0.688–1.020, *p* = 0.077). In the adjusted OS model, age (HR = 0.947, 95% CI: 0.886–1.012, *p* = 0.106), ECOG performance status (overall *p* = 0.165), and male sex (HR = 1.751, 95% CI: 0.758–4.046, *p* = 0.190) were not statistically significant. The gait-speed-by-log(time) interaction was not significant (HR = 0.758, 95% CI: 0.513–1.120, *p* = 0.164), providing no evidence of a proportional-hazards violation for the primary gait-speed effect (Table 3 and Table 4).

**Figure 3:**
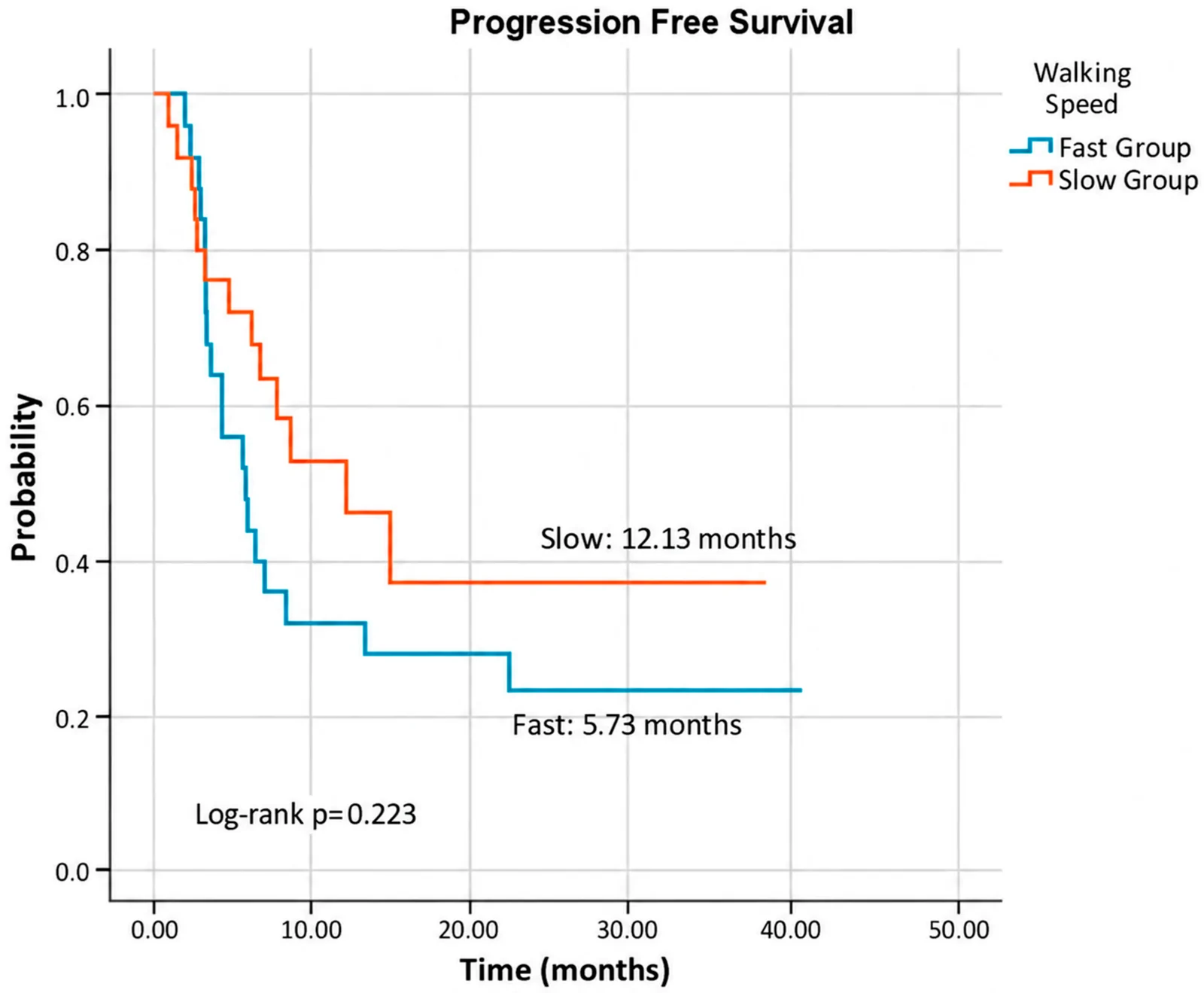
Kaplan–Meier Progression-Free Survival Curves in Metastatic Patients According to Gait Speed. Kaplan–Meier curves illustrating progression-free survival (PFS) in metastatic patients based on gait speed. Although the slow-walking group showed a numerically longer median PFS, the difference did not reach statistical significance (*p* = 0.223).

In the Kaplan–Meier analysis of PFS, median PFS was 5.73 months in the fast-walking group and 12.13 months in the slow-walking group. Although median PFS was numerically longer in the slow-walking group, the difference between the groups was not statistically significant (log-rank *p* = 0.223) (Figure 3).

### 3.3. Survival Analyses in Non-Metastatic Patients

Overall survival and disease-free survival (DFS) were analyzed according to gait speed. Although an approximate 7-month OS and 4-month DFS advantage was observed in the fast-walking group compared to the slow-walking group, these differences did not reach statistical significance (*p* = 0.301 and *p* = 0.805, respectively). The median OS was 19.7 months in females and 9.4 months in males (*p* = 0.010). In Kaplan–Meier analysis, disease-free survival was found to be significantly longer in patients with a history of surgery in non-metastatic disease (*p* = 0.017) (Table 5).

In non-metastatic patients, continuous gait speed was not significantly associated with OS in univariable analysis (HR = 0.955, 95% CI: 0.781–1.169, *p* = 0.658) or after adjustment for age, sex, and ECOG performance status (adjusted HR = 0.826, 95% CI: 0.652–1.047, *p* = 0.114). Likewise, gait speed was not associated with DFS in univariable analysis (HR = 1.141, 95% CI: 0.934–1.394, *p* = 0.195) or adjusted analysis (HR = 0.997, 95% CI: 0.803–1.236, *p* = 0.975). Male sex was associated with higher hazards in the adjusted OS (HR = 3.852, 95% CI: 1.575–9.420, *p* = 0.003) and DFS (HR = 3.413, 95% CI: 1.529–7.619, *p* = 0.003) models; given the limited subgroup size and heterogeneous diagnoses, this finding should be interpreted cautiously (Table 6 and Table 7).

### 3.4. Toxicity Assessment

**Figure 4:**
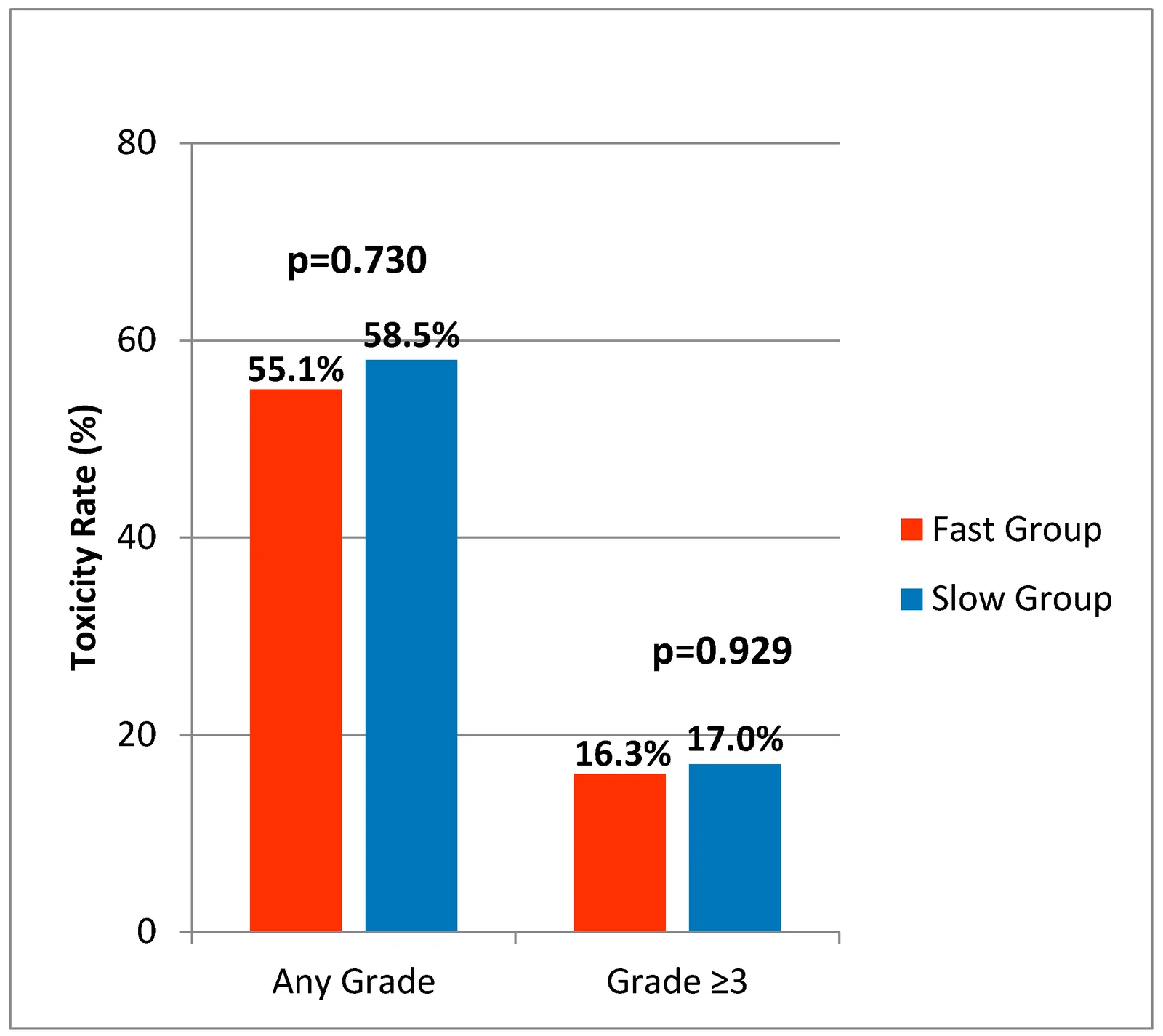
Comparison of Treatment-Related Toxicity Rates According to Gait Speed. Comparison of treatment-related toxicity rates between fast- and slow-walking groups. The incidence of any-grade toxicity and grade ≥ 3 toxicity was similar between the groups, with no statistically significant difference observed.

In the overall cohort, toxicity of any grade was observed in 56.9% (n = 58) of patients. The relationship between gait speed and the development of any toxicity was evaluated using the chi-square test. Toxicity occurred in 55.1% of the fast-walking group and 58.5% of the slow-walking group; however, this difference was not statistically significant (*p* = 0.730). No significant difference was found between the groups in terms of grade ≥ 3 severe toxicity (slow group: 17.0%, n = 9; fast group: 16.3%, n = 8; *p* = 0.929) (Figure 4).

When specific toxicity types were analyzed, different patterns were observed between hematologic and non-hematologic toxicities. No statistically significant association was found between gait speed and neutropenia levels (*p* = 0.996) or grade ≥ 3 neutropenia (*p* = 0.929).

Similarly, no significant associations were found between gait speed and thrombocytopenia (*p* = 0.241) or anemia (*p* = 0.824). Among non-hematologic toxicities, no significant association was observed between gait speed and nausea, vomiting, diarrhea, constipation, stomatitis, alopecia, fatigue, or asthenia (Table 8). These individual toxicity comparisons were exploratory. Grade ≥ 3 toxicity occurred in 17/102 patients (16.7%). In univariable logistic regression, continuous gait speed was not significantly associated with grade ≥ 3 toxicity (OR = 1.026, 95% CI: 0.765–1.377, *p* = 0.862). In the parsimonious model adjusted for age and sex, gait speed remained non-significant (OR = 0.999, 95% CI: 0.727–1.373, *p* = 0.995); age (OR = 1.029, 95% CI: 0.918–1.153, *p* = 0.624) and male sex (OR = 1.873, 95% CI: 0.522–6.720, *p* = 0.336) were also non-significant. Given the small number of grade ≥ 3 events, these estimates are exploratory and imprecise (Table 9).

In the 0.8 m/s sensitivity analysis, only 8/102 patients (7.8%) had gait speed ≥ 0.8 m/s, whereas 94/102 (92.2%) had gait speed < 0.8 m/s. In the metastatic subgroup, the corresponding numbers were 4/50 (8.0%) and 46/50 (92.0%). Using ≥0.8 m/s as the reference, gait speed < 0.8 m/s was associated with HR = 5.338 for metastatic OS, but the estimate was highly imprecise and not statistically significant (95% CI: 0.715–39.872, *p* = 0.103). The marked group imbalance limits interpretation of this sensitivity analysis.

## 4. Discussion

The objective measurement of functional capacity in the geriatric patient population is of critical importance in treatment decision-making and prognostic evaluation [8,9]. In the literature, gait speed has been shown to be a strong predictor of mortality, risk of falls, and loss of functional independence in the general population [10,13,14]. Prospective studies in geriatric oncology populations have also demonstrated associations between gait speed and clinical outcomes. Pamoukdjian et al. reported in a prospective cohort study of older cancer patients with a mean age of 80.6 years that usual gait speed below 0.8 m/s over 4 m was independently associated with early mortality within 6 months [15]. Similarly, in a multicenter analysis including 782 patients aged 75 years and older with hematological malignancies, Hantel et al. demonstrated that lower gait speed categories were associated with higher mortality risk in univariate analyses [16]. In older patients with acute myeloid leukemia (AML) receiving intensive induction chemotherapy, gait speed (as part of the SPPB) emerged as one of the strongest predictors of poor survival, and the addition of physical performance measures improved existing survival prediction models [17,21]. However, Aregui et al. reported that although gait speed was numerically associated with overall survival in metastatic non-small cell lung cancer patients receiving first-line systemic therapy, this association was not statistically significant [20].

In our study, higher continuous gait speed was remained associated with lower mortality in metastatic patients after adjustment for age, sex, and ECOG performance status. In contrast, no statistically significant association was detected between gait speed and metastatic PFS or the survival outcomes of the non-metastatic subgroup. Cancer type was not included in the Cox models because the number of tumor categories relative to subgroup size would increase model complexity; therefore, residual confounding by tumor type and disease setting cannot be excluded.

The continuous analysis suggests that better baseline mobility is associated with lower mortality in metastatic disease, whereas statistically significant associations were not detected for treatment-related grade ≥ 3 toxicity or other survival endpoints. These findings should be interpreted as exploratory associations rather than evidence that gait speed directly determines treatment tolerance or tumor response.

In the metastatic patient group, the multivariable analysis supported an association between continuous gait speed and OS after adjustment for age, sex, and ECOG performance status. Age, sex, and ECOG performance status were not statistically significant in the adjusted metastatic OS model. These findings should be interpreted cautiously given the limited subgroup size, heterogeneous tumor types, and potential residual confounding.

In the non-metastatic subgroup, continuous gait speed was not significantly associated with OS or DFS. Male sex was associated with higher hazards in the adjusted models, but this finding should be interpreted cautiously because of the limited number of events, heterogeneous tumor types, and the possibility of residual confounding. Mechanistic explanations for sex- or surgery-related differences cannot be established from the present data.

Grade ≥ 3 toxicity occurred in only 17 patients, and no statistically significant association with continuous gait speed was detected in either univariable or parsimonious adjusted logistic regression. This result should not be interpreted as proof that gait speed has no relationship with treatment tolerance, because the limited number of events reduces statistical power and precision. The individual toxicity comparisons were exploratory and involved multiple outcomes, increasing the possibility of chance findings. These findings are consistent with the conflicting results reported in the literature [16,17]. The limited ability of gait speed to predict toxicity may be explained by several factors. First, clinicians may adopt more conservative treatment approaches in frail patients by administering lower doses or less toxic regimens.

The strengths of our study include its prospective design, the use of a simple and easily applicable test in clinical practice, and the simultaneous evaluation of survival and toxicity outcomes. However, several limitations should be acknowledged. The study was single-center and included a limited number of patients with heterogeneous tumor types, disease settings, and treatment regimens. Regimen-level treatment intensity, dose modifications, supportive care, and subsequent therapies were not incorporated as covariates in the survival models and may contribute to residual confounding. The event counts, particularly in non-metastatic and toxicity analyses, limit statistical power and increase the risk of model instability or overfitting. Formal model calibration and discrimination comparisons were not performed. The sample-derived median cutoff entails information loss, while the 0.8 m/s sensitivity analysis was markedly imbalanced. Multiple toxicity comparisons were exploratory. No formal a priori sample-size calculation was performed, and functional mobility was assessed at a single baseline time point. Although both TUG and gait speed were assessed at baseline, the present analyses focused exclusively on separately measured gait speed; TUG was not included in the prognostic analyses.

## 5. Conclusions

In this exploratory prospective cohort, higher baseline gait speed remained associated with lower mortality in patients with metastatic cancer after adjustment for age, sex, and ECOG performance status. No statistically significant association was detected with metastatic PFS, non-metastatic OS or DFS, or grade ≥ 3 toxicity. Because of the limited sample size, heterogeneous tumor types and treatments, and restricted event counts for some outcomes, these findings require external validation before routine clinical implementation.

## Figures and Tables

**Table 1:** Baseline Patient Characteristics.

Characteristic	Fast Group	Slow Group	*p* Value
Age, years(mean ± SD)	70.3 ± 4.3	71.6 ± 5.0	0.168
Sex, n (%)			0.075
Female	13 (26.5)	23 (43.4)	
Male	36 (73.5)	30 (56.6)	
ECOG performance status, n (%)			0.446
0	9 (18.4)	12 (22.6)	
1	38 (77.6)	36 (68.0)	
2	2 (4.0)	5 (9.4)	
Smoking, n (%)			0.507
Current smoker	19 (41.3)	17 (34.7)	
Non-smoker	27 (58.7)	32 (65.3)	
Chronic Disease, n (%)			0.503
Yes	26 (56.5)	31 (63.3)	
No	20 (43.5)	18 (36.7)	
Metastasis Status, n (%)			0.698
Metastatic	25 (51.0)	25 (47.2)	
Non-metastatic	24 (49.0)	28 (52.8)	
Surgical History, n (%)			0.306
Yes	23 (46.9)	18 (36.7)	
No	26 (53.1)	31 (63.3)	
Diagnosis, n (%)			0.675
Lung cancer	16 (32.7)	16 (30.2)	
Breast cancer	8 (16.4)	7 (13.2)	
Gastrointestinal cancers	13 (26.5)	16 (30.2)	
Gynecologic cancers	3 (6.1)	6 (11.3)	
Other cancers *	9 (18.3)	8 (15.1)	

Continuous variables were compared using the independent-samples *t*-test, and categorical variables were compared using the chi-square test of independence. Missing data were present for smoking status (n = 7), comorbidity (n = 7), and surgical history (n = 4). Percentages for these variables were calculated using the number of patients with available data as the denominator. No imputation was performed for missing baseline data. * Other cancers include prostate cancer, bladder cancer, malignant melanoma, glioblastoma multiforme, squamous cell carcinoma of the skin, head and neck cancers, thymic carcinoma, and cancers of unknown primary origin.

**Table 2:** Kaplan–Meier Survival Analysis of Metastatic Patients According to Gait Speed and Clinical Variables.

Variable	Median OS (Months)	Log-Rank χ^2^	*p*-Value	Median PFS (Months)	Log-Rank χ^2^	*p*-Value
Walking speed (fast)	13.40 ± 2.30 [8.8–17.9] (95% CI)	4.465	0.035	5.73 ± 1.24 [3.2–8.1] (95% CI)	1.488	0.223
Walking speed (slow)	11.7 ± 1.46 [8.9–14.6] (95% CI)			12.13 ± 3.75 [4.7–19.4] (95% CI)		
ECOG 0	7.46 ± 0.91 [5.6–9.2] (95% CI)	5.228	0.073	6.26 ± 1.96 [2.4–10.1] (95% CI)	0.565	0.754
ECOG 1	13.03 ± 1.38 [10.3–15.7] (95% CI)			8.33 ± 1.68 [5.0–11.6] (95% CI)		
ECOG 2	14.83 ± 1.61 [11.6–18.0] (95% CI)			3.30		
Sex (female)	14.83 ± 1.35 [12.1–17.4] (95% CI)	0.000	0.997	6.26 ± 3.79 [0–13.7] (95% CI)	0.183	0.669
Sex (male)	11.7 ± 1.55 [8.7–14.8] (95% CI)			7.03 ± 1.36 [4.3–9.9] (95% CI)		
Smoking (no)	13.03 ± 1.83 [9.4–16.6] (95% CI)	1.760	0.185	8.66 ± 4.13 [0.5–16.7] (95% CI)	0.252	0.616
Smoking (yes)	12.86 ± 1.24 [10.4–15.2] (95% CI)			6.26 ± 1.32 [3.6–8.8] (95% CI)		
Chronic disease (no)	13.10 ± 1.77 [9.6–16.5] (95% CI)	0.042	0.837	8.33 ± 2.06 [4.2–12.8] (95% CI)	0.009	0.925
Chronic disease (yes)	12.86 ± 1.00 [10.9–14.8] (95% CI)			6.76 ± 1.88 [3.0–10.4] (95% CI)		
Surgery (no)	13.10 ± 1.04 [11.0–15.1] (95% CI)	1.410	0.235	6.76 ± 1.14 [4.5–9.0] (95% CI)	0.094	0.759
Surgery (yes)	8.36 ± 1.11 [6.1–10.5] (95% CI)			8.33 ± 7.01 [0–22.08] (95% CI)		

**Table 3:** Univariable Cox Regression Analyses of Continuous Gait Speed for OS and PFS in Metastatic Patients.

Outcome	Predictor	HR	95% Cl	*p*-Value
Overall survival (OS)	Gait speed, per 0.1 m/s increase	0.752	0.605–0.934	0.010
Progression-free survival (PFS)	Gait speed, per 0.1 m/s increase	0.846	0.703–1.017	0.075

Abbreviations: HR, hazard ratio; CI, confidence interval; OS, overall survival; PFS, progression-free survival; HRs for gait speed are reported per 0.1 m/s increase.

**Table 4:** Multivariable Cox Regression Analysis for Overall Survival and Progression-Free Survival in Metastatic Patients.

Outcome	Predictor	Adjusted HR	95% CI	*p*-Value
Overall survival (OS)	Gait speed, per 0.1 m/s increase	0.647	0.490–0.853	0.002
	Age, per year	0.947	0.886–1.012	0.106
	Male sex vs. female	1.751	0.758–4.046	0.190
	ECOG performance status (overall)	—	—	0.165
Progression-free survival (PFS)	Gait speed, per 0.1 m/s increase	0.838	0.688–1.020	0.077
	Age, per year	0.978	0.911–1.050	0.541
	Male sex vs. female	0.971	0.447–2.111	0.941
	ECOG performance status (overall)	—	—	0.846

Abbreviations: HR, hazard ratio; CI, confidence interval; OS, overall survival; PFS, progression-free survival; ECOG, Eastern Cooperative Oncology Group. HRs for gait speed are reported per 0.1 m/s increase.

**Table 5:** Kaplan–Meier Analysis for Overall Survival and Disease-Free Survival in Non-Metastatic Patients.

Variable	Median OS (Months)	Log-Rank χ^2^	*p*-Value	Median DFS (Months)	Log-Rank χ^2^	*p*-Value
Walking speed (fast)	19.73 ± 7.51 [5.0–34.4] (95% CI)	1.071	0.301	16.40 ± 8.47 [0–33.3] (95% CI)	0.061	0.805
Walking speed (slow)	12.46 ± 0.9 [10.7–14.2] (95% CI)			12.96 ± 2.88 [7.3–18.6] (95% CI)		
ECOG 0	12.96 ± 1.12 [10.7–15.1] (95% CI)	3.573	0.168	16.40	3.469	0.176
ECOG 1	15.56 ± 2.75 [10.1–20.9] (95% CI)			15.36 ± 3.39 [8.7–22.0] (95% CI)		
ECOG 2	7.86 ± 3.13 [1.7–14.0] (95% CI)			7.86 ± 3.56 [0.8–14.8] (95% CI)		
Sex (female)	19.73 ± 4.94 [10.0–29.4] (95% CI)	6.655	0.010	NR	10.929	0.001
Sex (male)	9.43 ± 4.67 [0.2–18.6] (95% CI)			4.23 ± 2.93 [0–9.9] (95% CI)		
Smoking (no)	16.40 ± 1.58 [13.2–19.5] (95% CI)	3.460	0.063	15.56 ± 2.62 [10.4–20.7] (95% CI)	1.628	0.202
Smoking (yes)	9.43 ± 4.24 [1.1–17.7] (95% CI)			4.23 ± 5.76 [0–15.5] (95% CI)		
Chronic disease (no)	16.40 ± 2.10 [12.2–20.5] (95% CI)	0.548	0.459	16.40 ± 2.56 [11.3–21.4] (95% CI)	0.122	0.727
Chronic disease (yes)	11.60 ± 0.89 [9.8–13.3] (95% CI)			10.33 ± 3.58 [3.3–17.3] (95% CI)		
Surgery (no)	16.40 ± 3.40 [9.7–23.0] (95% CI)	3.633	0.057	4.23 ± 2.87 [0–9.8] (95% CI)	5.660	0.017
Surgery (yes)	11.00 ± 4.89 [1.4–20.5] (95% CI)			21.70		

NR: Not reached.

**Table 6:** Univariable Cox Regression Analysis of Continuous Gait Speed for Overall Survival and Disease-Free Survival in Non-Metastatic Patients.

Outcome	Predictor	HR	95% CI	*p*-Value
Overall survival (OS)	Gait speed, per 0.1 m/s increase	0.955	0.781–1.169	0.658
Disease-free survival (DFS)	Gait speed, per 0.1 m/s increase	1.141	0.934–1.394	0.195

Abbreviations: HR, hazard ratio; CI, confidence interval; OS, overall survival; DFS, disease-free survival. HRs for gait speed are reported per 0.1 m/s increase.

**Table 7:** Multivariable Cox Regression Analysis for Overall Survival and Disease-Free Survival in Non-Metastatic Patients.

Outcome	Predictor	Adjusted HR	95% CI	*p*-Value
Overall survival (OS)	Gait speed, per 0.1 m/s increase	0.826	0.652–1.047	0.114
	Age, per year	0.958	0.864–1.063	0.422
	Male sex vs. female	3.852	1.575–9.420	0.003
	ECOG performance status (overall)	—	—	0.298
Disease-free survival (DFS)	Gait speed, per 0.1 m/s increase	0.997	0.803–1.236	0.975
	Age, per year	0.954	0.870–1.046	0.318
	Male sex vs. female	3.413	1.529–7.619	0.003
	ECOG performance status (overall)	—	—	0.225

Abbreviations: HR, hazard ratio; CI, confidence interval; OS, overall survival; DFS, disease-free survival; ECOG, Eastern Cooperative Oncology Group. HRs for gait speed are reported per 0.1 m/s increase.

**Table 8:** Treatment-Related Adverse Events According to Gait-Speed Group.

Adverse Event (Any Grade)	Fast Group, n (%)	Slow Group, n (%)	*p* Value
Hematologic toxicities			
Anemia	12 (24.5)	14 (26.4)	0.824
Neutropenia	12 (24.5)	13 (24.5)	0.996
Thrombocytopenia	6 (12.2)	3 (5.7)	0.241
Gastrointestinal toxicities			
Nausea *	18 (36.7)	20 (37.7)	0.917
Vomiting *	7 (14.3)	8 (15.1)	0.908
Diarrhea	3 (6.1)	2 (3.8)	0.583
Constipation	9 (18.4)	12 (22.6)	0.594
Stomatitis	6 (12.2)	2 (3.8)	0.112
Other toxicities			
Alopecia	8 (16.3)	7 (13.2)	0.657
Fatigue	17 (34.7)	14 (26.4)	0.364
Asthenia	2 (4.1)	0 (0)	0.137

* Prophylactic antiemetics were recommended but not mandatory. Categorical variables were compared using the chi-square test.

**Table 9:** Logistic Regression Analyses for Grade ≥ 3 Treatment-Related Toxicity in the Overall Cohort.

Model	Predictor	OR	95% CI	*p*-Value
Univariable	Gait speed, per 0.1 m/s increase	1.026	0.765–1.377	0.862
Multivariable	Gait speed, per 0.1 m/s increase	0.999	0.727–1.373	0.995
	Age, per year	1.029	0.918–1.153	0.624
	Male sex vs. female	1.873	0.522–6.720	0.336

Abbreviations: OR, odds ratio; CI, confidence interval. Grade ≥ 3 toxicity events: 17/102 (16.7%). The multivariable model was intentionally parsimonious because of the limited number of events.

## Data Availability

The datasets generated and/or analyzed during the current study are available from the corresponding author on reasonable request.
